# Complete Pathologic Response to PARP Inhibitor Olaparib in a Patient with Stage IVB Recurrent Endometrioid Endometrial Adenocarcinoma

**DOI:** 10.3390/jcm12113839

**Published:** 2023-06-04

**Authors:** Rosemary Noel Senguttuvan, Christina Wei, Mustafa Raoof, Thanh H. Dellinger, Edward Wenge Wang

**Affiliations:** 1 Department of Surgery, City of Hope Comprehensive Cancer Center (COH), Duarte, CA 91010, USA; rsenguttuvan@coh.org (R.N.S.); mraoof@coh.org (M.R.); tdellinger@coh.org (T.H.D.); 2 Department of Pathology, City of Hope Comprehensive Cancer Center (COH), Duarte, CA 91010, USA; cwei@coh.org; 3 Department of Medical Oncology, City of Hope Comprehensive Cancer Center (COH), Duarte, CA 91010, USA

**Keywords:** endometrial cancer, PARP inhibition, pathologic complete response, Olaparib, BRIP1 mutation, ATM mutation, RAD51C mutation, POLE mutation, TMB high

## Abstract

Treatment for endometrial cancer is rapidly evolving with the increased use and integration of somatic tumor RNA sequencing in clinical practice. There is a paucity of data regarding PARP inhibition in endometrial cancer given that mutations in homologous recombination genes are rare, and currently no FDA approval exists. A 50-year-old gravida 1 para 1 woman with a diagnosis of stage IVB poorly differentiated endometrioid endometrial adenocarcinoma presented to our comprehensive cancer center. Following surgical staging, she was placed on adjuvant chemotherapy with carboplatin/paclitaxel which was held multiple times due to poor performance status and complications. CT scan of the abdomen and pelvis following cycles 3 of adjuvant chemotherapy showed recurrent progressive disease. She received one cycle of liposomal doxorubicin but discontinued it due to severe cutaneous toxicity. Based on the BRIP1 mutation identified, the patient was placed on compassionate use of Olaparib in January 2020. Imaging during this surveillance period showed a significant decrease in hepatic, peritoneal, and extraperitoneal metastases, and eventually the patient had a clinical complete response in a year. The most recent CT A/P in December 2022 showed no sites of active recurrent or metastatic disease in the abdomen or pelvis. We present a unique case of a patient with recurrent stage IVB poorly differentiated endometrioid endometrial adenocarcinoma with multiple somatic gene mutations including BRIP1, who had a pathologic complete response following compassionate use of Olaparib for 3 years. To our knowledge, this is the first reported case of high grade endometrioid endometrial cancer that has shown a pathologic complete response to a PARP inhibitor.

## 1. Introduction

In 2022, an estimated 65,950 cases of uterine cancer were diagnosed in the United States with 12,550 women succumbing to the disease [1]. The incidence of uterine cancer has continued to rise in the United States over the last 10 years [1]. Certain factors known to negatively affect prognosis at the time of diagnosis include higher FIGO grade, extensive lymphovascular space invasion, invasion of the outer third of the myometrium, non-endometrioid histologic subtypes, and loss of p53 expression [2,3]. Treatment of early-stage disease involves upfront surgical staging with adjuvant chemotherapy and radiotherapy depending on final pathologic stage along with consideration of the presence of high-risk histological features. Chemotherapy and radiation following surgery for patients at advanced stages is recommended but carries significant short and long-term toxicities [3]. Thus, the investigation of alternative, less-toxic therapy is of interest.

Treatment for endometrial cancer is rapidly evolving with the increased uptake and integration of somatic tumor RNA sequencing and germline testing into clinical practice. The newly released 2023 National Comprehensive Cancer Network (NCCN) guidelines for uterine cancer include molecular tumor profiling into the four distinct molecular subtypes of endometrial cancer established by the Cancer Genome Atlas Research Network: (1) hypermutation in the exonuclease domain of DNA polymerase-ε (POLEmut); (2) mismatch repair deficiency, which confers microsatellite instability (MMRd); (3) mutations in TP53; and (4) tumors with none of the aforementioned classifications (‘no specific molecular profile’ or ‘NSMP’) [4,5]. Clinical trials are ongoing to determine the optimal treatment algorithm for each molecular category [6].

In ovarian cancer, the inhibition of poly (ADP-ribose) polymerase (PARP1) has resulted in superior clinical outcomes in patients that have germline or somatic mutations in BRCA1, BRCA2, and DNA homologous repair deficiency-related genes in frontline [7,8,9] and recurrent [10,11,12] settings. However, there is a paucity of data regarding use of PARP inhibition in endometrial cancer given that mutations in BRCA1, BRCA2 and other homologous recombination genes are rare [13]. There is currently no FDA approved use of PARP inhibitors in endometrial cancer. Various clinical trials are ongoing to elucidate the utility of PARP inhibitors in advanced or recurrent endometrial cancer, though data have yet to emerge [14,15].

We describe a case of a sustained complete pathologic response to Olaparib in a patient with stage IVB poorly differentiated endometrioid endometrial adenocarcinoma with multiple somatic mutations in the pathways of DNA repair, signal transduction, and metabolism.

## 2. Case Presentation

### 2.1. History of Presenting Illness

A 50-year-old gravida 1 para 1 woman with a diagnosis of stage IVB poorly differentiated endometrioid endometrial adenocarcinoma presented to the Gynecologic Oncology Department at City of Hope Comprehensive Cancer Center (COH) in mid-2019 for evaluation and treatment. Prior to presenting to COH, the patient had uterine leiomyomas diagnosed twenty years prior with subsequent menorrhagia, anemia, and multiple hospital visits. She had a negative endometrial biopsy on record from 2018. Following worsening bleeding in 2019, the patient underwent another endometrial biopsy, which identified a high-grade endometrial carcinoma. Pelvic ultrasound at this time showed the uterus with multilobulated appearance secondary to multiple leiomyomas (14 × 9.5 × 11 cm) with poor discernment of the endometrium. CT A/P without contrast prior to her presentation at COH showed a large multi-fibroid uterus (17.7 × 10.2 × 14.7 cm) and prominent retroperitoneal lymph nodes, progressively increased in size (18 mm × 15 mm), which is concerning for metastatic disease.

Her past medical history is significant for systemic lupus erythematosus, diagnosed at 16 years of age, and complicated with renal failure, requiring hemodialysis and renal transplant into the left lower pelvis at age 25, followed by long-term immunosuppression with prednisone. Her family medical history included a cousin with endometrial cancer of unknown age and was otherwise noncontributory. The patient had no history of smoking, alcohol, or drug use.

Physical exam at initial presentation was remarkable for a 20-week sized uterus. She was counseled regarding her diagnosis and elected to undergo primary surgery. She thus underwent an exploratory laparotomy, modified radical hysterectomy, bilateral salpingo-oophorectomy, right pelvic lymphadenectomy, and infra-gastric omentectomy in June 2019.

### 2.2. Surgical Findings

The patient had a globularly enlarged uterus measuring approximately 20 cm, with a tumor extending to the serosa, parametria, posterior lower uterine segment, and cervix, requiring a modified radical hysterectomy. The adnexa had no gross pathology. The posterior pelvic cul-de-sac was obliterated by the tumor. There were significant adhesions of the left pelvic kidney to the left lateral aspect of the uterus and parametrium, requiring extensive lysis of adhesions. An enlarged right common iliac lymph node was present. The omentum appeared grossly normal. The upper abdomen was within normal limits. At the end of the procedure, the patient was optimally cytoreduced.

### 2.3. Pathology

**Figure 1:**
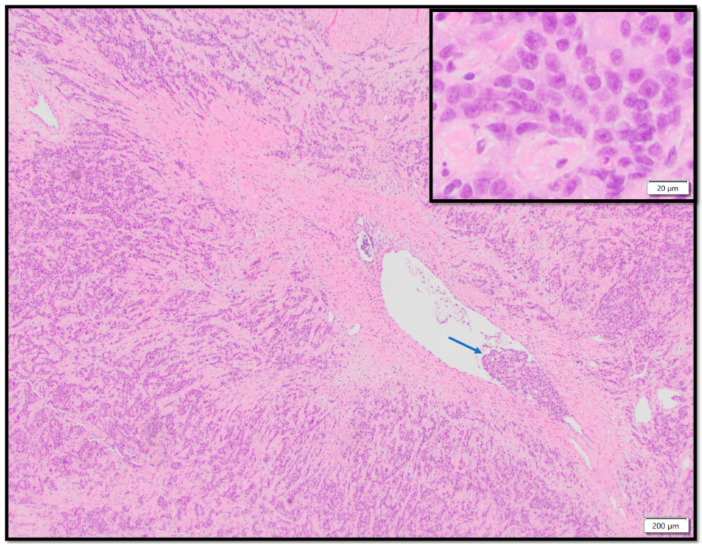
The histologic section of the uterine tumor showed poorly differentiated neoplastic cells with destructive growth pattern, diffusely infiltrating throughout the myometrium. The lower magnification image (4×) showed the presence of lymphovascular space invasion (arrow). The inset shows a high magnification view (40×) of the tumor cells, with high grade cytologic features including nucleomegaly, irregular nuclear contour, stippled to clumped chromatin, and variably prominent nucleoli. Scattered mitotic activity and numerous apoptotic cells can be seen, signifying high proliferative activity.

The hysterectomy specimen consisted of a 1251-g uterine corpus containing a 12.7 cm endometrial mass diffusely involving the myometrium, extending past the lower uterine segment into the anterior and posterior cervix. The tumor involved the left parametrium and cul de sac peritoneum, bilateral fallopian tubes and ovaries, and the omentum. Macrometastatic carcinoma (3.4 cm tumor deposit) was found involving the right common iliac lymph node. Microscopic examination of the endometrial tumor demonstrated a highly infiltrative, poorly differentiated carcinoma with extensive lymphovascular space invasion (Figure 1). Interestingly, tumor infiltrating lymphocytes were minimal in quantity and were not a prominent feature of the tumor. On balance, the immunomorphologic finding was consistent with a FIGO stage IV high-grade endometrial carcinoma, endometrioid type, FIGO grade 3. The patient was staged as stage IVB. An incidental serous tubal intraepithelial carcinoma was noted in one fallopian tube.

### 2.4. Immunohistochemistry (IHC)

The tumor immunoprofile demonstrated positivity for pan-cytokeratin, PAX8, and ER (60%, moderate intensity). TP53 IHC showed a wild-type staining pattern. Mismatch repair protein analysis demonstrated loss of nuclear expression for MSH6 and intact nuclear expression for MLH1, MSH2, and PMS2.

### 2.5. Somatic Tumor Testing

GEM ExTRA^®^ somatic tumor testing [16] was performed on the surgical specimen identifying alterations shown in Table 1, notably mutations in BRIP1 (c.2098-2A>G), ATM (R23*), RAD51C (R370*), POLE (V411L), MTOR (A469T), PTEN (R233*), TP53 (N131I), MSH2 (E580*), and MSH6 (E641*). The tumor had a high tumor mutation burden (344 Mut/Mb), but microsatellite instability (MSI) was stable. Germline testing was performed using Invitae Germline Precision Medicine American College of Medical Genetics (ACMG)^®^ Panels 22081I-VT0013 and 22081I-VT0014 and was uninformative [17].

When interpreted in conjunction with the molecular profile (POLE mutation p.V411L, high TMB, microsatellite stable), the molecular classification best fits the POLE-ultramutated molecular subtype in the context of a multiple-classifier (MSH6 deficiency by IHC, POLE mutation by molecular sequencing, and microsatellite stable status). The presence of TP53 mutation may be interpreted as a passenger mutation in this context.

### 2.6. Adjuvant Therapy and First Recurrence

Adjuvant chemotherapy with weekly carboplatin and paclitaxel was initiated but paused several times due to poor performance status and complications. CT scan of the abdomen and pelvis following three cycles of adjuvant chemotherapy showed recurrent progressive disease with new hepatic metastases, abdominopelvic peritoneal and mesenteric carcinomatosis, left supraclavicular, retroperitoneal, and pelvic nodal metastasis, and metastatic deposits within the abdominal wall. The patient was then switched to liposomal doxorubicin and received one cycle, but this was discontinued due to severe cutaneous toxicity necessitating hospitalization.

### 2.7. Compassionate Treatment with Olaparib

Based on the BRIP1 mutation identified, the patient was placed on compassionate use of Olaparib in January 2020, with the dose adjusted to 200 mg, twice daily. Imaging during this surveillance period showed a significant decrease in hepatic, peritoneal, and extraperitoneal metastases, and eventually the patient had a clinically complete response in a year.

The patient continued Olaparib until November 2022, when she experienced abdominal pain secondary to a ventral midline abdominal wall hernia with a resulting partial small bowel obstruction. After failed conservative treatment, the patient underwent a diagnostic laparoscopy with conversion to laparotomy, incisional/ventral hernia repair plus mesh implantation for recurrent obstruction and bowel incarceration within the hernia. Ileocecectomy/colectomy was also performed at this time due to plaque-like deposits noted on the serosal surface of the small bowel and mesentery, which are concerning for recurrent disease. Nodules in the omentum and transverse colon mesentery were resected. Pathology was negative for malignancy, and the transition point for the small bowel obstruction was thus determined to be due to scarring from the tumor treatment on the mesentery of the terminal ileum and right colon.

**Figure 2:**
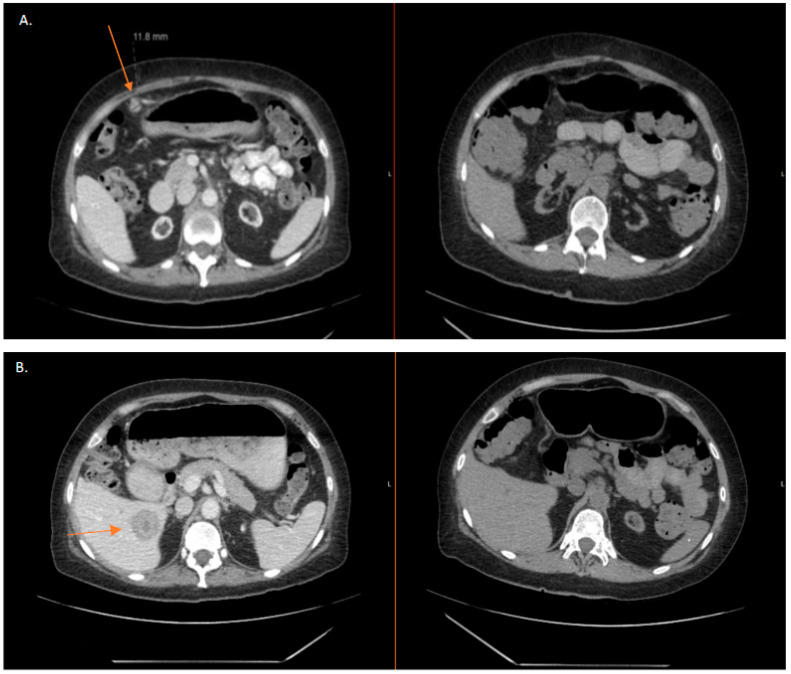
(**A**,**B**): CT comparison demonstrating regression of abdoinopelvic metastases represented by arrow. Axial views of metastases in October 2019 compared with corresponding axial views demonstrating complete radiographic regression of abdominopelvic metastases in December 2022.

The most recent CT A/P in December 2022 showed no sites of active recurrent or metastatic disease in the abdomen or pelvis. Figure 2A shows regression of peritoneal carcinomatosis by CT A/P at the time of progression in October 2019 in comparison to the most recent imaging in December 2022. Figure 2B is representative of the regression of one of the patient’s hepatic lesions. Though not shown, all other abdominal and hepatic metastases demonstrated complete regression. Olaparib was discontinued in January 2023, after 3 years. The patient is currently considered to have a complete response without radiographic and pathologic evidence of disease.

## 3. Discussion

We present a unique case of a patient with recurrent stage IVB poorly differentiated endometrioid endometrial adenocarcinoma with multiple somatic gene mutations including BRIP1, who had a pathologic complete response following compassionate use of Olaparib for 3 years. To our knowledge, this is the first reported case of high grade endometrioid endometrial cancer that has shown a pathologic complete response to a PARP inhibitor. One case of high grade serous endometrial carcinoma with clinical radiographic response to Olaparib is noted in the literature [18], however, no pathologic confirmation was reported.

PARP inhibition increases both progression-free survival and overall survival in patients with BRCA-deficient and homologous recombination deficient ovarian cancer [7,8,9,10,11,12]. Heeke and colleagues reported that 34.4% of endometrial cancers possess molecular aberrations of genes involved in the homologous recombination pathway [13]. BRIP1 (BRCA1 interacting helicase 1) is actively involved in the homologous recombination pathway by interacting with the BRCT repeats of BRCA1 [19]. A mutation of BRIP1 is associated with Fanconi anemia and breast cancer [20,21]; however, only 0.14% of endometrial cancers have BRIP1 mutations. This makes the study of BRCA-mutated and homologous recombination deficient mutated endometrial cancer patients difficult given the low incidence; however, it is worth investigation if clinical outcomes such as those reported in the ovarian cancer literature can be achieved with PARP inhibitor use in patients with endometrial cancer who harbor these germline and somatic mutations. There are ongoing clinical trials seeking to answer this question [14]. Other gene mutations in the DNA repair pathway including POLE, ATM, and RAD51C may also contribute to the significant response to PARP inhibition [22].

The classification of endometrial cancer is moving away from traditional type I and type II classification system and towards molecular categorization [4]. As the prognostic utility of molecular subtyping has been elucidated, the investigation of the optimal treatment for each of the four molecular subtypes originally determined by the Cancer Genome Atlas Research Network is ongoing in the overarching Refining Adjuvant Treatment In Endometrial Cancer (RAINBO) umbrella program [6]. This molecular classification works well for patients who neatly fit into each category; however, in cases such as ours where multiple genomic alterations exist that fit into more than one of the four molecular subcategories, the approach to treatment becomes challenging.

Our patient’s genomic analysis indicated her tumor was MMRd, POLE ultramutated, and possessed a passenger TP53 mutation with a resulting high tumor mutation burden. Given these multiple mutations, it is unclear which prognostic molecular subcategory she would fall into. This presents challenges to the clinician given the immense genomic heterogeneity, and an individualized approach to the specific molecular profile of the tumor should be undertaken to optimize clinical outcomes. Patients with endometrial cancer who have genomic alternations in homologous recombination related downstream genes should have a shared decision-making discussion with their provider regarding the potential benefits of PARP inhibitor therapy. Such decisions should be undertaken in collaboration with a multidisciplinary team consisting of the patient’s medical oncologist, surgeon, nursing team, and pharmacist to provide optimal team-based care.

## Figures and Tables

**Table 1:** Tumor genomic alterations identified by GEM ExTRA^®^ somatic tumor testing.

Gene Name	Gene Symbol	Mutation
AT-rich interactive domain-containing protein 1A	ARID1A	E1767*
ATM serine/threonine kinase	ATM	H1380Y
		R23*
		c.2921 + 1G>A
BRCA1 interacting protein C-terminal helicase 1	BRIP1	c.2098-2A>G
Cyclin-dependent kinase inhibitor 2A	CDKN2A	D74N
F-Box and WD repeat domain containing 7	FBXW7	R479Q
MutL homolog 3	MLH3	K703fs
MutS homolog 2	MSH2	E580*
MutS homolog 6	MSH6	E641*)
Mammalian target of rapamycin	MTOR	A469T
Neurofibromin 1	NF1	W221*
Phosphatidylinositol-4,5-bisphosphate 3-kinase catalytic subunit alpha	PIK3CA	E545D
		R348*
DNA polymerase epsilon catalytic subunit	POLE	V411L
Phosphatase and tensin homolog	PTEN	R233*
		T277A
RAD51 paralog C	RAD51C	R370*
Tumor protein P53	TP53	N131I
Tuberous sclerosis complex 2	TSC2	G1157*
Tumor mutational burden	TMB	High 344 mut/mB
Microsatellite instability	MSI	Stable

## Data Availability

Not applicable.
